# Management outcomes and factors related to hospital stay for pediatric intussusception in a Tertiary Care Hospital, Northwestern Ethiopia

**DOI:** 10.1371/journal.pone.0357668

**Published:** 2026-09-08

**Authors:** Nebiyu Shitaye Aniley, Worku Animaw Temesgen

**Affiliations:** 1 Department of Surgery, School of Medicine, College of Medicine and Health Sciences, Bahir Dar University, Bahir Dar, Ethiopia; 2 School of Nursing and Midwifery, College of Medicine and Health Sciences, Bahir Dar University, Bahir Dar, Ethiopia; Hacettepe University: Hacettepe Universitesi, TÜRKIYE

## Abstract

**Background:**

Intussusception is the most common cause of intestinal obstruction in infants and young children that requires prompt diagnosis and treatment to prevent complications and death. Nowadays, when cases are presented to hospitals on time and have no significant complications, non-invasive ultrasound-guided hydrostatic reduction is the first-line treatment, resulting in favorable outcomes, including higher recovery, lower complications, and shorter hospital stay compared to open surgery. However, evidence on management modalities, clinical outcomes, and factors related to hospital stay in resource-limited settings remains scarce. This study evaluated management outcomes and identified factors associated with the length of hospital stay among children with intussusception at a comprehensive specialized hospital in Northwestern Ethiopia, Bahir Dar.

**Methods:**

A retrospective study was conducted among pediatric patients diagnosed with intussusception at a comprehensive referral hospital between September 2020 and August 2024. Data were extracted from patient medical records using a structured data collection tool. Descriptive statistics were used to summarize patient characteristics and treatment outcomes. Non-parametric statistical tests, including Spearman’s correlation, Mann–Whitney U test, and Kruskal–Wallis test were used to examine the relationship between clinical variables and the length of hospital stay. Statistical significance was considered at *p* < 0.05.

**Results:**

A total of 257 children with intussusception were included in the study. The majority were males (62.6%), and nearly half (47.0) were rural residents. The mean age of participants was 18.8 months, while more than half of the cases (56.4%) were infants. Overall, 255 patients were successfully managed, while two died from complications related to late presentation. The majority (62.3%) were treated using ultrasound-guided hydrostatic reduction, while the rest required open surgical reduction. Most patients (91.8%) presented with ileocolic intussusception. Participants presented after prolonged symptom durations (mean 2.3 days), increasing the risk of complications. The majority had dehydration (67.6%), and over a quarter had fever at admission (28.0%). Longer hospital stay is associated with higher open surgical intervention (r = 0.89; p < 0.001); body temperature (r = 0.55; p < 0.001), elevated white blood cell count (r = 0.39; p < 0.001), longer duration of symptoms before presentation (r = 0.44; p < 0.001), number of hydrostatic reduction attempts (r = 0.54, p < 0.001), dehydration (η^2^ = 0.49; p < 0.001), and rural residency (r = 0.48; p < 0.001).

**Conclusion:**

The high burden of intussusception among children can be effectively managed with a non—operative hydrostatic reduction. However, late presentation, particularly from rural areas, remains a critical driver of complications and mortality. Strengthening early referral systems in peripheral health centers is essential to reducing the surgical burden and improving outcomes for children with intussusception.

## 1. Introduction

Intussusception is a significant, life-threatening surgical emergency characterized by the invagination of a proximal segment of the intestine into the lumen of an adjacent distal segment. It is recognized globally as the leading cause of intestinal obstruction in infants and toddlers, with varied clinical courses across geographic and socioeconomic contexts. Global intussusception estimates indicate a mean annual incidence that ranged from 34 (Africa) to 90 (Western Pacific Region) cases per 100,000 infants [1]. While it can occur across a broad pediatric age range up to 9 years, the vast majority of cases (approximately 60%) involve infants under 12 months, with over two-thirds occurring in males [2,3], peaking between 5 and 9 months of age [2].

In Low- and Middle-Income Countries (LMICs), particularly in Sub-Saharan Africa (SSA), the burden of intussusception and its complications is worsened by delayed presentation, inadequate infrastructure, and a lack of skilled personnel [4,5]. A recent 2025 systematic review reported that children in SSA often present with a mean symptom duration of over days, resulting in a pooled mortality rate of 12.4%, compared with less than 1% in high-income nations [6]. Delayed diagnosis and management allow intussusception to progress through venous and lymphatic obstruction, wall edema, and arterial insufficiency, culminating in intestinal necrosis, perforation, peritonitis, and septic shock [5]. In Ethiopia, over two-thirds of cases present to a healthcare setting after the critical three days [4,7]. This delayed presentation is associated with a high surgical burden, with disease advancement requiring bowel resection and anastomosis [4], accounting for over a quarter of complications, such as surgical infection and mortality [8]. Consequently, the intersection of late diagnosis and resource constraints transforms an otherwise treatable condition into a major contributor to childhood morbidity and mortality across Ethiopia and beyond [6].

Management of intussusception has evolved from mandatory laparotomy to the current gold-standard hydrostatic reduction (HR), which involves the retrograde administration of saline under ultrasound or fluoroscopic guidance to the bowel [5]. Although access to tertiary care settings offering this non-surgical option remains limited, HR offers a far safer and more efficient alternative to laparotomy with high success rates when performed early, reducing the surgical burden and hospital stays compared with the weeks often required after open surgery [8–11].

However, the successful implementation of HR requires a specific intersection of experts and infrastructure, conventionally assumed to require specially trained radiologists and pediatric surgeons who remain scarce in low-income settings [6–8,12]. As Ethiopia strives to expand pediatric surgical services and local expertise to reduce preventable childhood deaths [13], understanding the clinical outcomes and success rates of HR in local contexts is essential for guiding this expansion [14].

While HR is well established in high-income settings, its introduction in LMICs is comparatively recent, and adequate evidence describing the clinical characteristics, treatment modalities, and outcomes of intussusception management in these settings remains lacking. In Ethiopia specifically, no prior study has examined predictors of prolonged hospital stay or systematically compared outcomes between hydrostatic and surgical management, evidence that is essential for guiding earlier referral and improving resource allocation in similar low-resource settings. This study therefore aims to evaluate clinical presentations, demographic characteristics, and management outcomes of children with intussusception at a comprehensive specialized hospital in Ethiopia, and identify factors associated with the length of hospital stay, thereby contributing to local evidence to inform earlier referral pathways and expanded access to hydrostatic reduction services.

## 2. Methods

### 2.1 Study design, period, and setting

This study utilized a retrospective hospital-based design to evaluate the management and clinical outcomes of pediatric intussusception in children managed between 2020 and 2024. It was conducted at Tibebe Ghion Comprehensive Specialized Hospital, serving as a tertiary center for pediatric surgical emergencies. The hospital is one of the two comprehensive specialized teaching hospitals in the northwestern region of Ethiopia, primarily serving as the referral center for the southern and western catchment areas of the region.

The pediatric surgical service is among several other specialty-level clinical services the hospital is delivering. Before September 2019, all patients presenting with intussusception to hospitals in the northwestern region of the country were managed exclusively with open surgical intervention (laparotomy). The introduction of hydrostatic reduction of intussusception for the first time in the hospital in 2019, serving a population of over 10 million people in the region. Since its implementation, there has been a significant shift in practice, with nearly two-thirds of children with intussusception are now successfully treated using hydrostatic reduction, which is substantially improving patient outcomes.

### 2.2 Study population and participants

The study population consisted of pediatric patients up to the age of 14 years who presented to the emergency or pediatric departments with clinical symptoms indicative of intussusception (e.g., abdominal pain, vomiting, or “red currant jelly” stools). Participants were included in the study following a definitive diagnosis made through abdominal ultrasonography. The final decision regarding the clinical pathway, whether to attempt non-operative reduction or proceed directly to surgery, was determined by the clinical judgment of the attending pediatric surgeon based on the patient’s stability and duration of symptoms.

All children diagnosed with intussusception who presented to the hospital during the study period were evaluated and managed under the direct supervision of the team-leading senior physician, who prospectively recorded all cases. A consecutive sampling technique was employed to ensure all eligible cases during the study period were captured, eliminating the possibility of selection bias due to missed or unrecorded cases. This approach is consistent with prior studies of pediatric intussusception management in comparable settings [7,15]. A priori formal sample size calculation was therefore not applicable given the census-based design. Nonetheless, the resulting sample size (N = 257) exceeds the studies reported in comparable studies conducted in similar settings [7,15] and is adequate to support the bivariate statistical tests employed in this study [16].

### 2.3 Intervention modalities

Patients eligible for non-operative management underwent ultrasound-guided hydrostatic reduction ultrasound unit. Operating Room (OR) team was placed on standby to facilitate immediate surgery if complications, such as bowel perforation or failure, occurred. Decisions were made by surgical residents and approved by a pediatric surgeon based on duration of illness (<72 hours), vital signs, hydration status, abdominal physical findings, and sonographic results showing preserved blood flow. Parents gave informed consent after being briefed on the diagnosis, complications, treatment alternatives, and risks using visual aids.

Following intravenous access and fluid resuscitation at the pediatric emergency unit, patients were transferred to the ultrasound room and positioned supine. Warm normal saline was hung at a height of 120 centimeter above the level of the buttocks and a Foley catheter was inserted per rectum and inflated with 10–20 mL of normal saline. Saline was allowed to flow into the rectum through the catheter under real-time ultrasound guidance by a multidisciplinary team comprising radiologists, pediatric surgeons, and residents. No sedatives or anesthesia were administered to keep the patient conscious for early detection of complications. The entire procedure took approximately 15 minutes and could be repeated up to three times in 10-minutes intervals between [17]. After successful reduction, the patients were observed overnight with maintenance intravenous fluids and nil per os (NPO) status [18].

### 2.4 Data collection and analysis

Data on demographic, clinical, laboratory, intervention, and hospitalization details were collected from patient charts and procedural records via EpiCollect5 [19] between February 2 and 28, 2026, and exported to IBM SPSS (version 22) [20] for analysis. Descriptive analysis was done to summarize findings. Categorical variables are expressed as frequencies and percentages, and continuous variables are reported as means with standard deviations (SD) and presented using a table with appropriate narration in the results section. Because the length of hospital stay was not normally distributed, non-parametric tests, specifically the Mann–Whitney U test, Kruskal–Wallis test, and Spearman’s rank correlation, were used to analyze its bivariate relationships with categorical and continuous variables. Statistical significance was considered at p < 0.05.

### 2.5 Ethical considerations

This study utilized data extracted from patient medical records. Ethical approval for the use of these records was obtained from the Institutional Review Board (IRB) of Bahir Dar University under protocol number 6003/2026. The approval was originally granted for a broader research project that studies treatment outcomes of pediatric patients with surgical diagnoses at Tibebe Ghion Comprehensive Specialized Hospital. To ensure ethical compliance and protect patient privacy, the data used in this study are handled with strict confidentiality. No personal identifiers were collected during the data extraction process. Specifically, identifying information such as the names of the index child, parents, or guardians was not recorded. All data were analyzed in an anonymized form and were used solely for research purposes.

## 3. Results

### 3.1 Sociodemographic and clinical characteristics

**Fig 1:**
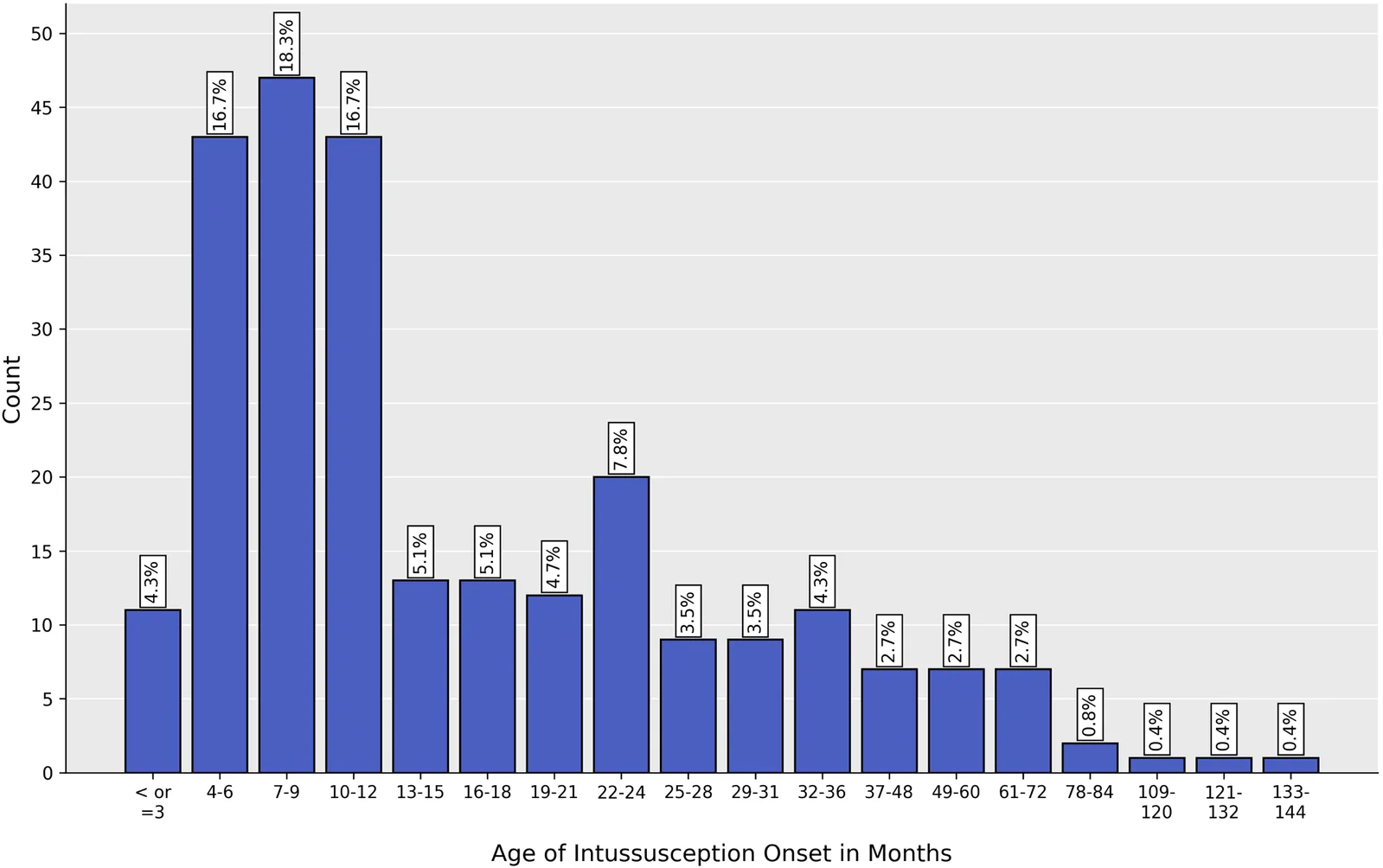
Incidence of Intussusception and its distribution by age at TGCSH during study period (N = 257).

In this report, 257 children who have presented to Tebebe Ghion Comprehensive Specialized Hospital (TGCSH) between September 2020 and August 2024 with complaints that led to an intussusception diagnosis have participated. The majority of the study participants were males, 62.6% (n = 161). In terms of residency, the distribution was nearly even, with 52.5% living in urban areas and 47.0% in rural areas. The mean age of the participants was 18.8 months (range: 1–144 months). Nearly two-thirds (56.4%) of the intussusception cases occurred within the first year of life. Specifically, infants aged 3 months and below account for 4.3% (N = 11) and infants aged 6 months or younger accounts 21.4% (N = 55) of cases. As children grow older, the frequency stabilizes at a lower rate. Less than a quarter (22.2%) of cases occurred in children aged 1–2 years, with the remaining 21.4% occurring in children aged 2 years and above. Fig 1 depicts the distribution of intussusception among age groups.

Regarding medical history, a minority of patients had a history of upper respiratory infection (13.2%) or gastroenteritis (12.8%) a few days before presentation, and only a very small fraction (1.6%) had experienced a previous episode of intussusception. The patients presented after a mean symptom duration of 55.2 hours (range: 3–240 hours). Upon admission, the majority of patients exhibited some level of dehydration, with 51.8% categorized as having some dehydration and 16.7% suffering from severe dehydration. While 45.1% of patients presented with a normal temperature of 36.0°C, nearly one-third showed signs of fever (axillary temperature 38° C and above). The mean weight of participants at presentation was 11.4 kg. Anatomically, ileo-colic intussusception was the most common type, accounting for 91.8% of cases. Laboratory findings showed a mean hemoglobin level of 11.4 g/dl and a mean WBC count of 11,112.7 cells/mm³, although counts varying from 3,010–27,020 cells/mm³. Table 1 presents the details of these figures.

**Table 1:** General characteristics of participants (N = 257).

Variable	Category	Freq (%)	Hydrostatic Reduction Freq (%) 160 (62.3)	Open Surgery Freq (%) 97 (37.7)
Sex	Male	161 (62.6)	99 (38.5)	62 (24.1)
	Female	96 (37.4)	61 (23.7)	35 (13.6)
Residence	Urban	135 (52.5)	115(44.8)	20(7.9)
	Rural	121 (47.0)	45(17.5)	76(29.6)
History of upper respiratory infection	Yes	34 (13.2)	22(8.6)	12(4.7)
	No	223 (86.8)	138(53.7)	85(33.1)
History of Gastroenteritis	Yes	33 (12.8)	19(7.4)	14(5.4)
	No	224 (87.2)	141(54.9)	83(32.3)
Previous History of Intussusception	Yes	4 (1.6)	1(0.4)	3(1.2)
	No	253 (98.4)	159(61.9)	94(36.3
Location of intussusception	Colo-colic	11 (4.3)	5(1.9)	6(2.3)
	Ileo-colic	236 (91.8)	155(60.3)	81(31.5)
	Ileo-ileo-colic	3 (1.2)	–	3(1.2)
	Ileo-colo-colic	5 (1.9	–	5(1.9)
	Small bowel to small bowel	2 (0.8)	–	2(0.8)
Hydration Status at presentation	No dehydration	81 (31.5)	77(30.0)	4(1.6)
	Some dehydration	133 (51.8)	83(32.3)	50(19.5)
	Severe dehydration	43 (16.7)	–	43(16.7)
Axillary Temperature in Celsius at presentation	36.0	116(45.1)	101(39.3)	15(5.8)
	37.0	69(26.8)	50(19.5)	19(7.4)
	38.0	53(20.6)	9(3.5)	44(17.1)
	39.0	18(7.0)	–	18(7.0)
	40.0	1(0.4)	–	1(0.4)
	Min-Max	Mean with SD	Mean with SD	Mean with SD
Age in months	1-144	18.8(19.7)	19.4(14.9)	17.8(25.7)
Length of hospital stay in days	1-24	3.4(4.3)	1.2(2.0)	7.0(4.6)
Weight in KG	4.00-36.00	11.4 (4.8)	11.7(3.7)	10.0(6.1)
Duration of symptom in hrs until presentation	3:00-240	55.2 (45.8)	33.5(27.4)	90(47.6)
Hemoglobin in g/dl	7.0-16.0	11.4(1.9)	11.6(1.7)	11.0(2.1)
WBC	3,010-27020	11,112.7 (4086.1)	9383.1(2863.4)	13851.3(4414.8)

### 3.2 Intussusception management modalities and treatment outcomes

Among all study participants, 99.2% (255 of 257) were successfully managed, while 2 (0.8%) died. The majority of successfully managed cases were treated by hydrostatic intervention (160, 62.3%), and the remainder by open surgical intervention (95, 37.0%). The two deceased children were both managed by open surgery and were among the 15 postoperative complications. They were from rural areas, one male and one female, aged 5 and 9 months, respectively, and presented too late, 5 and 7 days after the onset of clinical symptoms, with gangrenous bowel and peritonitis. Both children presented with multiple complication signs including, severe dehydration, bloody/jelly stool, high-grade fever (both 39 degrees Celsius), high WBC counts (17500 and 20840), and low hemoglobin (7 and 8 g/dl). Despite surgical interventions to remove gangrenous tissue, one child died a day after arrival, and the other died after 21 days of hospitalization following repeated laparotomy.

Patients in this study followed two distinct routes depending on the presenting conditions of the intussusception. For eighty-nine of these children, a decision was made to immediately proceed to open surgery at the first outset for the conditions they presented with not eligible for hydrostatic reduction. The remaining 168 children began with ultrasound-guided hydrostatic reduction. While this was successful for the vast majority on the first attempt (145 cases), 23 children required a second attempt, and 15 eventually moved to a third. By the end of this process, 160 (95.2%) children were successfully treated without an incision. However, for 8 children, even three consecutive attempts at hydrostatic reduction could not reduce the intussusception, leading them eventually to open surgical management.

When comparing children managed by hydrostatic reduction with those managed by open surgery, several clinical differences were evident. Hydration status at presentation differed substantially between groups: children with no dehydration were overwhelmingly managed by hydrostatic reduction (95.1%), whereas all children with severe dehydration underwent open surgery. A similar pattern was observed for axillary temperature, with children presenting at higher temperatures (39–40°C) managed exclusively by open surgery. Children managed by hydrostatic reduction had a substantially shorter mean length of hospital stay (1.2 ± 2.0 days) than those managed by open surgery (7.0 ± 4.6 days) and presented earlier with a shorter mean duration of symptoms before presentation (33.5 ± 27.4 hours) compared with the open surgery group (90.0 ± 47.6 hours).

The eight cases who required surgical management after failed hydrostatic reduction attempts presented with ileocolic intussusception primarily involving males (6 out of 8) with a mean age of 17 months. These patients presented with a mean symptom duration of 54.7 hours and displayed stable vital signs and laboratory results. Notably, clinical presentation included dehydration in all except one. Abdominal and physical examinations revealed different findings. Three patients had no relevant abdominal signs, three presented with a palpable rectal mass, and two demonstrated blood on digital rectal examination. Despite the failure of non-operative reduction, all these cases were successfully managed by open surgical intervention, with every patient recovering fully and being discharged after a hospital stay of 3–5 days.

**Fig 2:**
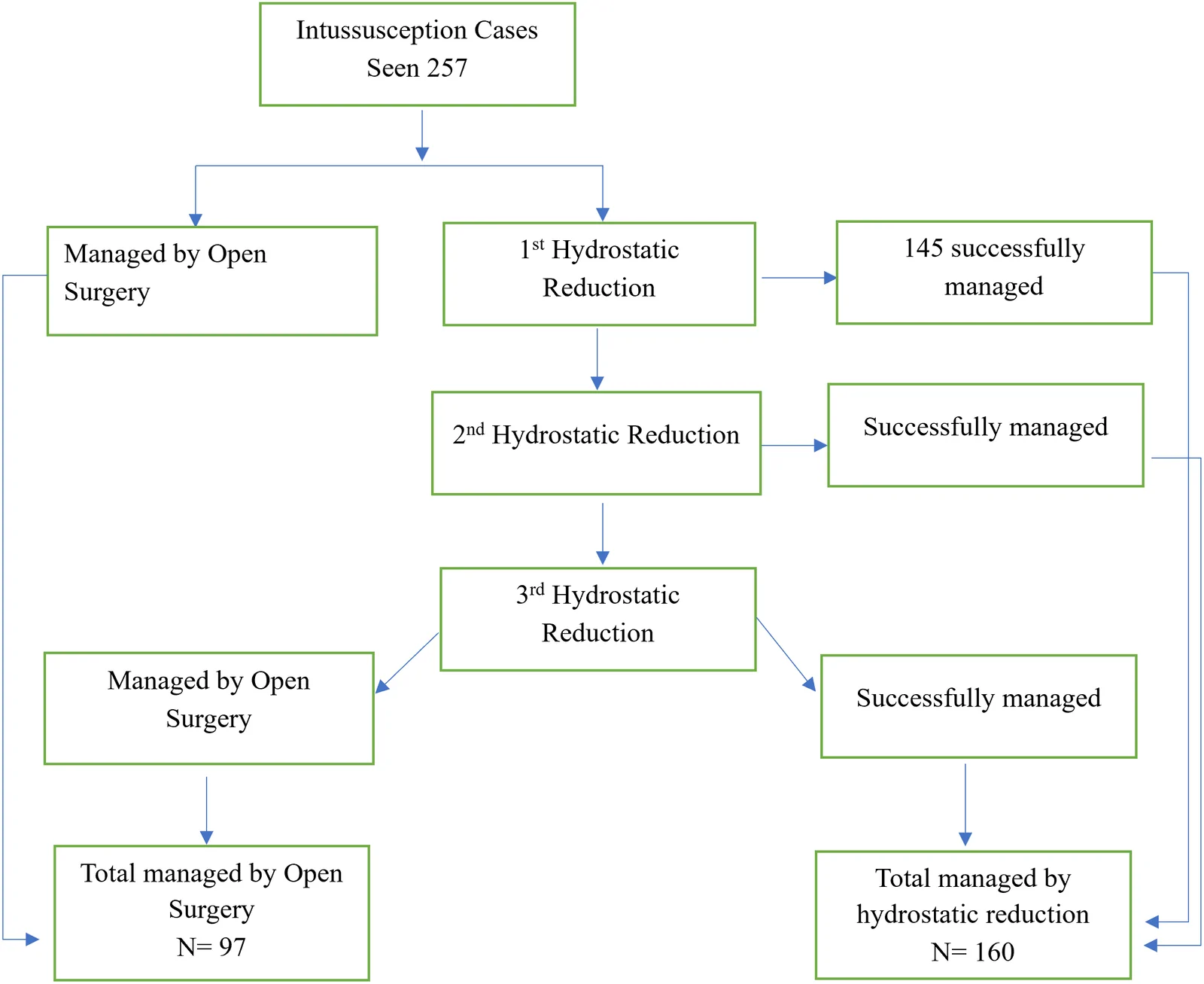
Flow diagram for intussusception management modalities age at TGCSH during study period (N = 257).

For children managed by open surgery (N = 97), over half (50.5%) were found to have gangrenous bowel tissue, with 41.2% requiring resection and anastomosis and 7.2% needing a stoma. In contrast, 42.3% of intussusceptions were viable and managed through manual reduction alone, while 8.2% of operations involved removal of a specific pathological lead point to prevent future recurrence. In one case, the intussusception had already subsided spontaneously by the time the operation began. Among children managed by open surgery (N = 97), the majority, 82 (84.5%), experienced an uncomplicated recovery. The remaining (15, 15.5%) developed postoperative complications such as wound infection (5, 5.2%), wound infection with pneumonia (4, 4.1%) and re-laparotomy for wound dehiscence, anastomotic leak and abdominal abscess (4, 4.1%), and two died despite relaparotomy. Fig 2 presents the details of patient flow.

### 3.3 Length of hospital stay and related factors

The mean length of hospital stay for management of intussusception in pediatrics was 3.4 days. A majority of patients (61.1%) were discharged within 24 hours, while 26.8% remained hospitalized for 2–7 days, and 12.1% required an extended stay of more than one week. Table 1 presents details of these figures.

Spearman’s rho correlation test was run to assess the statistical relationship between selected variables and the length of stay. Several physiological, clinical, and demographic characteristics are significantly associated with length of hospital stay. Body temperature showed the strongest positive correlation with length of hospital stay (r = 0.55, p < 0.001). Similarly, white blood cell (WBC) count demonstrated a moderate positive correlation with the length of hospital stay (r = 0.39, p < 0.001). Duration of symptoms before presentation showed a moderate positive correlation with hospital stay (r = 0.44, p < 0.001). The number of hydrostatic reduction trials showed a strong positive correlation with the length of hospital stay (r = 0.54, p < 0.001). Younger children and children with lower body weight tend to have longer hospital stays (r = –0.28 for age and r = –0.22 for weight, p < 0.001 for both). Hemoglobin level showed a weak negative correlation with hospital stay (r = –0.19, p < 0.001), suggesting that lower hemoglobin levels may be associated with longer hospitalization. Table 2 presents the length of hospital stay for intussusception management and its relation with these variables.

**Table 2:** Length of hospital stay, its distribution, and correlation among groups.

Variable	Spearman’s rho r(p-value) (r = correlation coefficient, p = 2 tailed sig value)						
	Duration of Symptom	Weight	Temperature	Hemoglobin	WBC	Number of hydrostatic trials	Length of stay
Age	.03 (0.7)	.74 (<0.001)	−.16 (0.01)	.33 (<0.001)	−.02 (0.70)	−.18 (0.24)	−0.28(<0.001)
Duration of Symptom	1	.03(0.67)	.42 (<0.001)	−.19(0.002)	.32 (<0.001)	.09 (0.08)	.44 (<0.001)
Weight		1	−.24 (<0.001)	.44 (<0.001)	−.14 (0.02)	.06 (0.42)	−.22 (<0.001)
Temperature			1	−.28 (<0.001)	.51 (<0.001)	.09 (0.21)	.55 (<0.001)
Hemoglobin				1	−.23 (<0.001)	−.01 (0.9)	−.19 (<0.001)
WBC					1	−.05 (0.5)	.39 (<0.001)
Number of hydrostatic trials						1	.54(<0.001)
Variable	Category			Length of stay in days Median (IQR)		Mann-Whitney U Test r(p-value)	
Sex	Female			1.0(3.8)		0.01 (0.82)	
	Male			1.0(3.0)			
Residence	Rural			3.5(6.0)		0.48(<0.001)	
	Urban			1.0(0.0)			
Intervention Type	Hydrostatic			1.0(0.0)		0.89(<0.001)	
	Surgical			6.0(4.0)			
Variable	Category			Length of stay in days Median (IQR)		Kruskal-Wallis H Test η^2^ (p-Value)	
Hydration Status	No dehydration			1.0(0.0)		0.49(<0.001)	
	Some dehydration			1.0(2.5)			
	Severe dehydration			8.0(8.0)			

Table 2 presents a comparison of the median length of hospital stay across categorical variables among children treated for intussusception using Mann–Whitney U and Kruskal–Wallis H tests. Females and males have nearly similar hospital stay (p = 0.82). Children from rural areas had a significantly longer median hospital stay (3.5 days) compared with those from urban areas (1.0 days) (p < 0.001). Children who underwent hydrostatic reduction had a substantially shorter median hospital stay (1.0 days) than those who required surgical intervention (6.0 days) (p < 0.001). Children with no dehydration had the shortest median hospital stay (1.0 days) as compared with those with severe dehydration (8.0 days) p < 0.001), indicating that worsening dehydration status is strongly associated with prolonged hospitalization.

Overall, these correlation findings suggest that clinical severity indicators, particularly higher temperature, elevated WBC count, longer symptom duration before treatment, and the need for repeated hydrostatic reduction attempts, are associated with prolonged hospital stay among children treated for intestinal intussusception. In addition, residence, type of intervention, and hydration status are important determinants of the length of hospital stay in children with intestinal intussusception.

## 4. Discussion

This study assessed the sociodemographic and clinical characteristics of children with intussusception diagnosed and managed at a comprehensive specialized hospital in a low-income African setting, including Ethiopia. It further evaluated the clinical outcomes of ultrasound-guided hydrostatic and open surgical reduction, particularly the success rate and length of hospital stay, and identified factors associated with these outcomes. In this study, 257 children, the majority (62.6%) male and with a mean age of 18.8 months, were diagnosed with intussusception over four years, highlighting the continued burden of this condition in pediatric surgical practice. Previous studies have documented that males are approximately twice as likely as females to develop intussusception. Similar male predominance has been reported in studies from both high-income and low- and middle-income countries [1,6]. However, the underlying reasons for male predominancy remain unclear [1,21].

The majority (56.4%) of intussusception cases in this study occurred in infants, with the highest magnitude observed between seven and twelve months, which is in line with several national [4,8,15] and international [1,6] studies. The higher incidence in this age group is thought to be related to rapid intestinal growth, lymphoid hyperplasia associated with viral infections, and changes in feeding patterns, which may predispose children to intestinal telescoping [22,23]. Recognizing the prominence of disease in this age group underscores the importance of safe, minimally invasive interventions, as these young patients are less able to tolerate the physiological stress and postoperative recovery associated with conventional surgery.

The mean duration of symptoms before hospital presentation was 55.2 hours, indicating that many children presented relatively late after symptom onset. Delayed presentation is known to significantly affect the success of management, increase the risk of complications, and affect the overall outcome. Studies have demonstrated that less invasive procedures, such as hydrostatic reduction, are most successful when performed within the first 24–48 hours of symptom onset. In contrast, delayed presentation is associated with high failure rates and the need for open surgical management, causing more complications. [15,24,25]. A large proportion of patients presented with fever and dehydration due to persistent vomiting, reduced oral intake, and fluid sequestration in the obstructed bowel, which could be due to delayed presentation and later contributed to delayed intervention and prolonged hospital stay [9].

Regarding the management modalities used and outcomes, the majority (99.2%) of patients in this study were successfully treated, either through ultrasound-guided hydrostatic reduction (62.3%) or open surgical intervention (37.7%). Two patients in the present study unfortunately died (0.8%) due to late presentation with advanced disease complications, including bowel gangrene, peritonitis, severe dehydration, high fever, leukocytosis, and severe anemia. However, this mortality rate is remarkably low compared with previous reports. In previous studies, the death rate due to intussusception among admitted patients was high in Africa (10.0%) [1] and Ethiopia (13.0%) [4]. This significant variation can be attributed to the study setting. The current study was conducted in a tertiary hospital equipped with highly skilled personnel, advanced equipment, hydrostatic reduction service, and a standby surgical team. Notably, hydrostatic reduction was introduced to the region in late 2019, and the hospital is the only center providing this service in the western region and one of the two in northwestern Ethiopia. Outcomes might have differed significantly had the study been conducted before service implementation. Studies from low-resource settings have consistently shown that limited healthcare access, delayed referral systems, and lack of early recognition contribute significantly to poor outcomes [1,26].

All cases successfully managed by ultrasound-guided hydrostatic reduction recovered without sequela and were discharged after a short hospital stay, whereas eight patients required open surgery following three failed consecutive hydrostatic attempts. This finding supports existing evidence that non-operative reduction remains an effective first-line treatment for pediatric intussusception when patients present early and without complications. Previous studies have reported hydrostatic reduction success rates ranging from 60% to over 93%, depending on the timing of presentation, patient condition, and available expertise [10,12,15,24]. In this study, hydrostatic reduction achieved a high overall success rate of 95.2% among attempted cases, outperforming previous Ethiopian reports from Addis Ababa (81.6%) [4] and Bahir Dar (93.1%) [15]. These findings suggest that expanding ultrasound-guided reduction service could substantially reduce the need for surgical intervention. It is widely preferred over laparotomy because it is significantly less invasive and carries a much lower morbidity rate for pediatric patients. This method avoids surgical trauma on an infant’s developing immune system, drastically reducing the incidence of postoperative ileus and the formation of lifelong peritoneal adhesions that can lead to future bowel obstructions [23]. The success of hydrostatic reduction observed in this study highlights the importance of early diagnosis and the availability of ultrasound-guided intervention in improving outcomes in resource-limited settings.

Despite the success of non-operative management, a considerable proportion (37.7%) of children in this study required open surgical intervention. Delayed presentation remains the primary factor driving the necessity of surgery and adverse outcomes in pediatric intussusception [27]. The mean duration of symptoms before presentation was 55.2 hours, indicating that many children arrived past the optimal 48-hour window for successful non-operative reduction [24,25,28]. Similar findings have been reported across several low- and middle-income countries, where delayed health-seeking behavior, weak referral systems, and long travel distances contribute to late hospital presentation [9,21,29]. Prolonged symptom duration leads to persistent vomiting, severe dehydration, bowel fluid sequestration, ischemia, and necrosis, which significantly increases the failure rate of hydrostatic reductio [30,31]. The two deaths observed in this study further illustrate the tragic consequences of delayed presentation with advanced complications (gangrenous bowel, peritonitis, and sepsis), reinforcing previous evidence that delayed diagnosis and treatment are major predictors of poor outcomes in pediatric intussusception [32,33].

Another important finding of this study was the strong association between clinical severity indicators and duration of hospital stay. Children presenting with higher body temperature, elevated white blood cell count, prolonged symptom duration, and severe dehydration experienced significantly longer hospitalizations. Fever and leukocytosis reflect underlying intestinal inflammation or ischemia, both of which complicate management and delay recovery. Severe dehydration presented in a large proportion of patients, resulting from persistent vomiting and fluid sequestration [29]. These findings highlight the critical need for prompt fluid resuscitation, temperature control, and physiologic stabilization during early emergency care.

Rural residence was another major factor associated with longer hospital stays compared with urban residence (median 3.5 vs 1.0 days, P < 0.001). Rural living is frequently linked to limited health care access, delayed referral pathways, and lower caregiver awareness, leading to more advanced disease at presentation [27,32]. Similar urban-rural disparities in outcomes have been documented in other low-resource settings [1,8]. Given that the majority of the Ethiopian population resides in rural areas, addressing these systematic barriers is essential to reduce the burden of advanced intussusception.

Overall, these findings reinforce the paramount importance of early diagnosis and timely management in improving outcomes for children with intussusception. Expanding access to non-operative ultrasound-guided hydrostatic reduction, improving primary care triaging, and enhancing clinical awareness among healthcare providers and caregivers could significantly reduce surgical intervention, prevent complications, and shorten hospital stays. Together, the predominance of cases among infants, the high success rate of hydrostatic reduction, and the severe risks associated with delayed presentation underscore the urgent need to strengthen pediatric emergency care and referral systems in resource-limited settings.

### 4.1 Limitations

The study has several limitations in population coverage and method. The study was conducted at a single tertiary hospital, which limits the generalizability of the findings to other settings. The retrospective nature of medical record review may limit the completeness and comprehensiveness of available data. Some potentially relevant variables, such as family-related factors, early in-hospital or post-discharge recurrence, complications, and long-term consequences, were not considered in this study. Consequently, the reported success rate of hydrostatic reduction reflects initial procedural resolution rather than overall recurrence-free recovery. Finally, due to the nature of the data, the analysis was limited to bivariate relationship tests only.

## 5. Conclusion and recommendations

The significant burden of intussusception among children under one year of age in Ethiopia underscores the urgent need for prompt, minimally invasive interventions to avoid the physiological stress of conventional open surgery. This study confirms that ultrasound-guided hydrostatic reduction is highly effective and significantly reduces both morbidity and hospital stay. However, the high incidence of complications and deaths linked to delayed presentation, particularly among rural populations. This suggests the critical need to strengthen early referral systems and expand access to appropriate treatment in resource-limited settings. Based on the findings, the study team recommends training first-line health workers on early symptom recognition, streamlining referral pathways, and expanding ultrasound-guided hydrostatic reduction to other lower-tier hospitals across Ethiopia. Furthermore, the team recommends that future research adopt a multi-center, prospective design that incorporates family-related factors, post-discharge surveillance, and data suitable for inferential statistical testing. The caregiver care-seeking journey—including their psychosocial and mental health experiences warrant further qualitative exploration triangulated with prospective findings, to better understand the derivers contributing to delayed presentation and to inform context-specific interventions for timely care.
