# Supplementary material for: Management outcomes and factors related to hospital stay for pediatric intussusception in a Tertiary Care Hospital, Northwestern Ethiopia
Source: PLoS One. 2026 Sep 8;21(9):e0357668. doi: 10.1371/journal.pone.0357668 (PMC13552730; doi:10.1371/journal.pone.0357668)
Supplement: Supplementary file 1 [file pone.0357668.s002.docx]

**14/Aug/2026**

**Dear Academic Editor**

**Re:** Submission of Revised Manuscript “Management Outcomes and Factors Related to Hospital Stay for Pediatric Intussusception in a Tertiary Care Hospital, Northwestern Ethiopia”

We are pleased to submit the revised version of our manuscript for consideration for publication. We express our sincere gratitude to the Academic Editor and both Reviewers for their rigorous and insightful evaluation of our work. In response to their comments, we have thoroughly revised the manuscript particularly, tightening the introduction and methods sections, enhancing the depth and flow of the Discussion section, clarifying study limitations, and incorporating context-specific recommendations. Please have a look at the table for a comprehensive, point-by-point response to all comments. We have attached two versions of the manuscript for your review: a clean revised version and a version with tracked changes highlighting all edits.

Thank you for your time and continued consideration of our work.

Sincerely,

Worku A. Temesgen

On behalf of all authors

| **Comments by Academic Editor** | Response |
| --- | --- |
| 1. The manuscript would benefit from a clearer statement of its specific contribution to the existing literature. The authors should revise the hypothesis and objective to emphasize what this study adds beyond existing evidence, such as local management outcomes, predictors of prolonged hospital stay, or implications for earlier referral and access to care. | We thank the Academic Editor for this valuable comment. We have revised the final paragraphs of the Introduction to more clearly articulate the specific contribution of this study to the existing literature. Specifically, we now state that while hydrostatic reduction is well established in high-income settings, its introduction in Ethiopia and similar low-resource settings is comparatively recent, and adequate evidence describing the clinical characteristics, treatment modalities, and outcomes of intussusception management in these settings remains lacking. We further clarify that this study contributes evidence on predictors of prolonged hospital stay and a comparison of outcomes between hydrostatic and surgical management within the Ethiopian context, which is intended to inform earlier referral pathways and expanded access to hydrostatic reduction services in similar resource-limited settings. This revised framing, along with the corresponding revision to the study objective, can be found on pages 4 (final two paragraphs of the Introduction). |
| 2. The abstract should provide the corresponding statistical values, particularly p values and/or confidence intervals, for these associations. | We thank you for this comment. The corresponding statistical values, including p-values and/or correlation coefficients for these associations, have been added to the Abstract in the revised manuscript (Page 2 -3, Lines 39-43). |
| 3. The Introduction is somewhat lengthy and could be streamlined. | We thank the Academic Editor for this comment. We have significantly revised the Introduction by condensing background information that was already well established, streamlining the discussion of intussusception pathophysiology and management history, and focusing more directly on the specific knowledge gap, rationale, and public health relevance of delayed presentation and preventable mortality in this setting. The revised Introduction is shorter and more focused, while retaining the essential background needed to contextualize the study's contribution (pages 3–4). |
| 4. Figure 1 appears blurred and should be replaced with a higher-resolution version | We thank the Academic Editor for this comment. We have replaced Figure 1 with a higher-resolution version (TIFF format), meeting PLOS ONE's figure specifications and substantially improving readability of the age distribution data. |
| 5. Figure 3 may be omitted or revised to improve clarity. | We thank the Academic Editor for this comment. We have removed Figure 3 from the manuscript, retaining the corresponding findings as a narrative description within the Results text. This addresses the reviewer's related concern regarding inconsistent percentage values between the figure and the text |
| **Comments by reviewer 1** |  |
| 1. Introduction is too long and the aim of the study has not been clearly described. (comment NO 3 above) 2. Methods section is also too long. | We thank the reviewer for this comment. We have significantly revised the Introduction and Methods sections by condensing their contents, removing redundant and tangential background information, and sharpening the focus of both sections. The revised Introduction now clearly states the study's aim and specific contribution in its closing paragraphs (pages 3–4), and the Methods section has been streamlined to remove duplicated and extraneous detail (pages 5–7) |
| 3. The protocol of the study is not evidence-based.   How did they perform 3 consecutive attempts?  Definition of successful hydrostatic reduction?  Indications for hydrostatic reduction  NPO to minimize the risk of early recurrence (2 hours after successful HR is recommended in the latest literature)   4. The protocol of the study is not evidence based. | We sincerely thank the reviewer for this important and constructive comment. We carefully reviewed the relevant literature and revised the Methods section to provide greater clarity regarding our institutional protocol for ultrasound-guided hydrostatic reduction. Specifically, the indications for non-operative management were clarified, the sonographic criteria for successful reduction were defined, and the protocol for repeated reduction attempts, including the 10-minute interval between attempts and the circumstances under which further attempts were undertaken.  We also incorporated relevant published evidence regarding repeated reduction attempts and post-reduction management. In particular, previous clinical experience has described the use of repeated saline-enema reduction attempts in the management of ileocolic intussusception as mentioned somewhere at Flaum V. et al., 2016.  Concerning post-reduction fasting, we acknowledge the reviewer's important observation that more recent evidence supports earlier feeding after successful non-operative reduction, including a shorter period of NPO. The overnight NPO practice described in the original manuscript reflected our institution's existing intussusception management protocol during the study period and can also be supported by older references [Al-Jazaeri A. et al., 2006] rather than a recommendation based on current evidence. We have therefore revised the wording in the Methods to present this explicitly as an institutional post-procedure observation practice rather than as a measure proven to prevent early recurrence.  Furthermore, in response to this comment, we are initiating a multidisciplinary review of our hospital's intussusception management guideline, with the aim of updating the protocol in accordance with current evidence, including recommendations regarding post-reduction feeding and observation.  The relevant references have been added to the revised manuscript: |
| 5. Although the authors mentioned the lack of recurrence data as a limitation, however recurrence is an important outcome if the researchers want to analyze the success ratio of hydrostatic reduction. | We thank the reviewer for this critical comment regarding recurrence as a key outcome when evaluating hydrostatic reduction. We recognize that recurrence can occur both early during the initial hospital stay (e.g., within 24–48 hours post-reduction) and later post-discharge.  Because this was a retrospective medical record review, complete and standardized capture of early in-hospital recurrent episodes following initial successful hydrostatic reduction was limited by variable chart documentation. Consequently, the scope of this study was defined around the primary index reduction attempt and overall in-hospital outcomes (immediate non-operative success vs. surgical conversion, total length of stay, and mortality).  We fully agree with the reviewer that tracking both early in-hospital and late post-discharge recurrence is essential for comprehensively evaluating the durability of hydrostatic reduction. In response, we have updated the limitations section to explicitly acknowledge this constraint. Furthermore, we have strengthened our Conclusion and Recommendations to emphasize the need for future prospective studies equipped with standardized monitoring protocols for both early in-hospital recurrences and prospective post-discharge follow-up.  (Page 15 Line 382-402) |
| 6. Discussion section is not adequate and superficial. | We express our sincere gratitude for this valuable feedback regarding the depth of our Discussion section. In response, we have comprehensively restructured and expanded the Discussion section to move beyond descriptive statistics, offering a deeper synthesis of our clinical findings, pathophysiological considerations, and the health-system implications of pediatric intussusception management. Specifically, we contextualized our numerical results; such as management success rates, clinical severity indicators, and rural-urban disparities, within broader national and international literature to ensure a rigorous, insightful interpretation of the data. These updates are located throughout the revised **Discussion** section (Page 16-18, Paragraphs 3–7). |
| **Comments by reviewer 2** |  |
| 1. This is an important study that assesses factors associated with hospital stay and outcome of intussusception patients in Northern region in Ethiopia. It highlights the importance of non-invasive methods such as hydrostatic reduction in patient management and emphasizes key gaps and recommendations to improve timely care. | We thank the reviewer for this positive assessment of our study and its relevance to improving timely care for pediatric intussusception in Ethiopia. We address each specific comment below. |
| 2. Overall, the manuscript is good and addresses a relevant topic. However, it needs some revision and a thorough grammar review to improve academic rigor. Particularly, the correct use of tense, and use of casual words such as “besides” and repeated use of “also” is not fit for academic writing and should be corrected/replaced. | The manuscript has undergone a thorough language edit throughout the document, with correction of tense inconsistencies and removal/replacement of casual phrasing including 'besides' and repeated use of 'also'. |
| 3. Please include location (Ethiopia/Northwestern Ethiopia) in the title and abstract. | The Title and Abstract have been revised to specify the study location (Ethiopia — Northwestern Ethiopia) Bahir Dar.”  (page 1; line 3 and page 2; 5 line 22).  Now the title reads as   “Management Outcomes and Factors Related to Hospital Stay for Pediatric Intussusception in a Tertiary Care Hospital, Northwestern Ethiopia**”** |
| 4. The information in line 114 regarding the population served by the hospital is repeated in lines 126–127. Consider removing the earlier statement. Additionally, clarify whether this is the only comprehensive hospital serving the region or if there are other comparable hospitals. | We have removed the earlier, less specific statement and retained the more detailed description. We have also clarified that the Tibebe Ghion Comprehensive referral hospital is one of the two (with University of Godar Hospital) comprehensive specialized teaching hospitals in Northwestern Ethiopia, primarily serving as the referral center for the southern and western catchments (page 5 line 117-119). |
| 5. Are there any articles/references to support the statement on how laparotomy was associated with longer stay and higher morbidity at the center? If it was meant in a more general sense, it also needs to be rephrased and appropriate references included. | We thank the reviewer for this comment. This statement is supported by existing literature documenting higher complication rates and prolonged recovery associated with surgical management of pediatric intussusception in resource-limited settings, including in the Ethiopian context specifically [Molla et al., 2025; Wakjira et al., 2018]. We have added these citations at the relevant point in the manuscript. In addition, this pattern is directly demonstrated by our own findings, in which children managed by open surgery had a substantially longer mean length of hospital stay (7.0 ± 4.6 days) compared with those managed by hydrostatic reduction (1.2 ± 2.0 days), as presented in the Results section. |
| 6. Use of abbreviations such as ICU, OR etc. first must be spelled in full description at the first mention. | All abbreviations (including ICU, OR, and others) have been either spelled out or replaced with their full descriptions at first mention throughout the manuscript and consistently used thereafter. (Page 5, Line 121) |
| 7. Include a sample size or justification. | We thank the reviewer for this comment. We would like to clarify that no sub-sampling was performed in this study. A consecutive sampling technique was used specifically to capture every eligible case presenting during the study period, meaning this represents a complete census of intussusception cases managed at the site during this time, rather than a sample drawn from a larger pool. This is further supported by the fact that all evaluations and treatments (both hydrostatic reduction and laparotomy) during the study period were performed and recorded by the team-leading senior physician, who is also a co-author and the primary source of this dataset, minimizing the possibility of missed or unrecorded cases. This approach is consistent with prior descriptive and factor-association studies of pediatric intussusception management in comparable Ethiopian tertiary/comprehensive specialized hospital settings, which similarly relied on consecutive or institution-based enrollment with smaller cohorts (n = 103 and n = 145, Zewde et al., 2024; Ayana et al., 2025 rspectively. Furthermore, the resulting sample size (N = 257) is adequate to support the bivariate statistical tests employed in this study (Mann–Whitney U, Kruskal–Wallis, and Spearman's rank correlation tests) [Serdar et al., 2021], and robustly addresses the study objective of evaluating clinical presentations, demographic characteristics, management outcomes, and factors associated with length of hospital stay among children with intussusception at a comprehensive specialized hospital. We have revised the Methods section to state this explicitly (page 5-6, lines 113-128) and have clarified that a formal sample size calculation was not applicable given the census-based design. |
| 8. Information on factors affecting decision on mode of treatment is duplicated and should be removed. | Yes, the comment is absolutely correct, and the section under 2.2 is deleted. |
| 9. The last paragraph in the demographics subsection describes treatment modality and outcome and should be included in the second subsection on management and outcome. | Thank you; now it is revised for language and moved to the next sub-section |
| 10. Fig. 2. Flow diagram, on the left side where open surgery numbers are reported, instead of total managed by open surgery it written as total managed by hydrostatic reduction, needs to be corrected. | Thank you again for picking up this typographical error. We have corrected the label in Fig. 2 (Flow Diagram); the box on the left side previously misstated as "Total managed by hydrostatic reduction" now correctly reads "Total managed by open surgery" in the revised manuscript. |
| 11. Fig.3. Are the numbers percentages? If so, it needs to be written with % or mentioned in the description. Additionally in the graph it says 82% no complication while 83.5 % is used in the result section. Findings should be consistent and as well as the use of decimals throughout the manuscript. | We thank the reviewer for this comment. Rather than revise Figure 3, we have opted to remove and present the corresponding findings as a narrative description within the Results text as suggested by academic editor. This resolves both the ambiguity regarding whether the values represented percentages and the inconsistency between the figure (82%) and the Results text (83.5%), as the finding is now reported once, in text, using the verified value. |
| 12. Remove "it" from subsection title page 278. | Addressed on Page 10 line 251 |
| 13. Table 2 is confusing and seems like two separate tables merged. | We thank the reviewer for this comment. We considered splitting Table 2 into separate tables; however, we have opted to retain it as a single table, as it presents a unified set of bivariate analyses examining the relationship between patient variables and length of hospital stay; the specific statistical test differs by variable type (Spearman's rank correlation for continuous variables; Mann-Whitney U for binary categorical variables; Kruskal-Wallis H for multi-level categorical variables), but all serve the same analytic purpose. To improve clarity and address the concern that the table appeared to merge unlike contents, we have added a labeled header row above the correlation matrix (consistent with the existing headers for the Mann-Whitney and Kruskal-Wallis sections) so that all three components of the table are clearly and consistently demarcated. In addition, as the data were analyzed using nonparametric tests, we have revised the summary statistics for length of hospital stay from mean (SD) to median (IQR) throughout the table, to appropriately reflect the underlying distribution and align with the statistical methods used. Please see Table 2 |
| 14. It would be great to have a table that summarizes findings with hydrostatic reduction vs open surgery. | We thank the reviewer for this helpful suggestion that significantly enhances the manuscript.  We have added a new cross-tabulation to Table 1, presenting the distribution of variables by treatment modality (hydrostatic reduction vs. open surgery) alongside the original overall frequencies. Please see the revised Table 1 and described. (Page 9 line 220-228) |
| 15. Discussion... repetition... merge paragraphs. | We sincerely thank the reviewer for this insightful comment regarding the repetition and structure across lines 333 and 380. In response, we have thoroughly consolidated these sections into a single, cohesive discussion paragraph addressing symptom duration, surgical necessity, and clinical outcomes, while performing significant language and grammatical edits throughout the section. Specifically, the repetitive figures and overlapping narrative have been eliminated to enhance conciseness and clarity. These revisions can be found in the revised manuscript under the **Discussion** section.  (Page 13-14) |
| 16. Conclusion and Recommendations It’s great you included points on how findings relate to broader low resource setting and SDGs, but you first need to highlight the implications of findings and specific recommendations for Ethiopian context.  When drafting the recommendations, please avoid using directive language such as "shall be." Instead, frame the recommendations as suggestions based on the study findings, for example, "Based on the findings, the study team recommends…". You have highlighted several important gaps for future research.  Additionally, I recommend emphasizing the need for qualitative studies exploring caregivers' experiences which would provide a deeper understanding of the factors contributing to delayed presentation and help inform context-specific interventions to improve timely care. | We sincerely thank the reviewer for these practical and constructive suggestions to strengthen our Conclusion and Recommendations. In response, we replaced all directive phrasing with non-directive, evidence-based phrasing (*"Based on the findings, the study team recommends..."*), expanded context-specific recommendations for expanding pediatric emergency care in Ethiopia, and explicitly highlighted the need for future qualitative studies exploring caregivers' experiences regarding delayed presentation. These additions can be found in **Conclusion and recommendations section**,  Page 15; lines 392–406). |
