# Supplementary material for: Management outcomes and factors related to hospital stay for pediatric intussusception in a Tertiary Care Hospital, Northwestern Ethiopia
Source: PLoS One. 2026 Sep 8;21(9):e0357668. doi: 10.1371/journal.pone.0357668 (PMC13552730; doi:10.1371/journal.pone.0357668)
Supplement: Peer review history

# Peer review: Decision Letter 0

**DOI:** 10.1371/journal.pone.0357668.r001

**Authors:** Ozlem Boybeyi

20 Jul 2026

Dear Dr. Temesgen,

Thank you for submitting your manuscript to PLOS ONE. After careful consideration, we feel that it has merit but does not fully meet PLOS ONE’s publication criteria as it currently stands. Therefore, we invite you to submit a revised version of the manuscript that addresses the points raised during the review process.

==============================

**ACADEMIC EDITOR:**

The manuscript addresses the management outcomes of pediatric intussusception in a tertiary care hospital and includes a sizeable cohort of 257 patients. The study addresses an important and clinically meaningful issue, especially in relation to delayed presentation and preventable mortality in pediatric intussusception. The topic is clinically relevant, particularly from a global public health perspective. However, the manuscript would benefit from a clearer statement of its specific contribution to the existing literature. The authors should revise the hypothesis and objective to emphasize what this study adds beyond existing evidence, such as local management outcomes, predictors of prolonged hospital stay, or implications for earlier referral and access to care.

==============================

Please submit your revised manuscript by Sep 03 2026 11:59PM. If you will need more time than this to complete your revisions, please reply to this message or contact the journal office at plosone@plos.org. When you're ready to submit your revision, log on to https://www.editorialmanager.com/pone/ and select the 'Submissions Needing Revision' folder to locate your manuscript file.

- A letter that responds to each point raised by the academic editor and reviewer(s). You should upload this letter as a separate file labeled 'Response to Reviewers'.
- A marked-up copy of your manuscript that highlights changes made to the original version. You should upload this as a separate file labeled 'Revised Manuscript with Track Changes'.
- An unmarked version of your revised paper without tracked changes. You should upload this as a separate file labeled 'Manuscript'.

If you would like to make changes to your financial disclosure, please include your updated statement in your cover letter. Guidelines for resubmitting your figure files are available below the reviewer comments at the end of this letter.

If applicable, we recommend that you deposit your laboratory protocols in protocols.io to enhance the reproducibility of your results. Protocols.io assigns your protocol its own identifier (DOI) so that it can be cited independently in the future. For instructions see: https://journals.plos.org/plosone/s/submission-guidelines#loc-laboratory-protocols. Additionally, PLOS ONE offers an option for publishing peer-reviewed Lab Protocol articles, which describe protocols hosted on protocols.io. Read more information on sharing protocols at https://plos.org/protocols?utm_medium=editorial-email&utm_source=authorletters&utm_campaign=protocols.

As the corresponding author, your ORCID iD is verified in the submission system and will appear in the published article. PLOS supports the use of ORCID, and we encourage all coauthors to register for an ORCID iD and use it as well. Please encourage your coauthors to verify their ORCID iD within the submission system before final acceptance, as unverified ORCID iDs will not appear in the published article. *Only*  the individual author can complete the verification step; PLOS staff *cannot*  verify ORCID iDs on behalf of authors.

We look forward to receiving your revised manuscript.

Kind regards,

Ozlem Boybeyi

Academic Editor

PLOS One

Journal requirements:

When submitting your revision, we need you to address these additional requirements.

1. Please ensure that your manuscript meets PLOS ONE's style requirements, including those for file naming. The PLOS ONE style templates can be found at

https://journals.plos.org/plosone/s/file?id=wjVg/PLOSOne_formatting_sample_main_body.pdf and

https://journals.plos.org/plosone/s/file?id=ba62/PLOSOne_formatting_sample_title_authors_affiliations.pdf

If the reviewer comments include a recommendation to cite specific previously published works, please review and evaluate these publications to determine whether they are relevant and should be cited. There is no requirement to cite these works unless the editor has indicated otherwise.

Additional Editor Comments:

The manuscript addresses the management outcomes of pediatric intussusception in a tertiary care hospital and includes a sizeable cohort of 257 patients. The study addresses an important and clinically meaningful issue, especially in relation to delayed presentation and preventable mortality in pediatric intussusception. The topic is clinically relevant, particularly from a global public health perspective. However, the manuscript would benefit from a clearer statement of its specific contribution to the existing literature. The authors should revise the hypothesis and objective to emphasize what this study adds beyond existing evidence, such as local management outcomes, predictors of prolonged hospital stay, or implications for earlier referral and access to care.

Besides this and the recommendations of the reviewers, the following issues should also be revised:

The abstract should provide the corresponding statistical values, particularly p values and/or confidence intervals, for these associations. Without these data, it is difficult for readers to assess the strength and reliability of the reported findings.

The Introduction is somewhat lengthy and could be streamlined. The authors should consider shortening background information that is already well established and focus more directly on the rationale for this specific study, the knowledge gap, and the public health relevance of delayed presentation and preventable mortality.

Figure 1 appears blurred and should be replaced with a higher-resolution version to improve readability.

Figure 3 may be omitted or revised to improve clarity.

[Note: HTML markup is below. Please do not edit.]

Reviewers' comments:

Reviewer's Responses to Questions

**Comments to the Author**

1. Is the manuscript technically sound, and do the data support the conclusions?

Reviewer #1: Yes

Reviewer #2: Yes

**********

2. Has the statistical analysis been performed appropriately and rigorously?

Reviewer #1: Yes

Reviewer #2: Yes

**********

3. Have the authors made all data underlying the findings in their manuscript fully available?

The PLOS Data policy

Reviewer #1: Yes

Reviewer #2: Yes

**********

4. Is the manuscript presented in an intelligible fashion and written in standard English?

Reviewer #1: No

Reviewer #2: No

**********

Reviewer #1: 1. Introduction is too long and the aim of the study has not been clearly described. There is too much additional information, that makes hard to focus on the topic.

2. Methods section is also too long and involves too much additional information, that can be discussed in another section.

3. protocol of the study is not evidence based.

for example:

how did they perform 3 consecutive attempts

definition of successful hydrostatic reduction

indication for hydrostatic reduction

NPO to minimize the risk of early recurrence (2 hours after successful HR is recommended in latest literature)

4. Although the authors mentioned the lack of recurrence data as a limitation, however recurrence is an important outcome if the researchers want to analyze the success ratio of hydrostatic reduction.

5. Although there have been detailed statistical analysis of data, discussion section is not adequate and superficial.

Reviewer #2: This is an important study that assesses factors associated with hospital stay and outcome of intussusception patients in Northern region in Ethiopia. It highlights the importance of non-invasive methods such as hydrostatic reduction in patient management and emphasizes key gaps and recommendations to improve timely care.

Overall, the manuscript is good and addresses a relevant topic. However, it needs some revision and a thorough grammar review to improve academic rigor. Particularity, the correct use of tense, and use of casual words such as “besides” and repeated use of “also” is not fit for academic writing and should be corrected/replaced accordingly. Please find below my specific comments and suggestions.

Please include location (Ethiopia/ Northern- Western Ethiopia) in the title and abstract

Methods

The information in line 114 regarding the population served by the hospital is repeated in lines 126–127. Consider removing the earlier statement, as the latter provides more specific information. Additionally, it would be helpful to clarify whether this is the only comprehensive hospital serving the region or if there are other comparable hospitals, as this would provide important context.

Are there any articles/references to support the statement on how laparotomy was associated with longer stay and higher morbidity at the center? If it was meant in more a general sense also needs to be rephrased and appropriate references included.

Use of abbreviations such as ICU, OR etc. first must be spelled in full description at the first mention with the abbreviations in parentheses, then the abbreviated form can be used for the remainder.

Include a sample size or justification. The use of consecutive sampling is appropriate for this study design; however, providing an estimated sample size based on the number of patients seeking care at the study site would strengthen the statistical validity of the study

Information on factors affecting decision on mode of treatment (Open Vs Hydrostatic reduction) in study population section is duplication as it is mentioned in intervention modality section, would remove.

Result

The last paragraph in the demographics sub-section describes treatment modality and outcome of patients so it should be included in the second sub-section on management and outcome.

Fig. 2. Flow diagram, on the left side where open surgery numbers are reported, instead of total managed by open surgery it written as total managed by hydrostatic reduction, needs to be corrected.

Fig.3. Are the numbers percentages? If so, it needs to be written with % or mentioned in the description. Additionally in the graph it says 82% no complication while 83.5 % is used in the result section. Findings should be consistent and as well as the use of decimals throughout the manuscript.

Remove “it” from sub-section title page 278

Table 2 is confusing and seems like two separate tables merged, needs to be clearer and adjusted.

It would be great to have a table that summarizes findings with hydrostatic reduction vs open surgery of the different variables assessed (days before arrival/duration of symptoms, length of stay, status on arrival (temperature, hydration etc.).

Discussion

Discussion is good and highlights critical findings in the study. However, there is repetition of points and needs to be more concise and grammatically edited. Particularly the paragraph starting from line 333 and line 380, are both discussing points related to delayed presentation, better to merge and highlight the main points.

Conclusion and Recommendations

It’s great you included points on how findings relate to broader low resource setting and SDGs, but you first need to highlight the implications of findings and specific recommendations for Ethiopian context.

When drafting the recommendations, please avoid using directive language such as "shall be." Instead, frame the recommendations as suggestions based on the study findings, for example, "Based on the findings, the study team recommends…". You have highlighted several important gaps for future research.

Additionally, I recommend emphasizing the need for qualitative studies exploring caregivers' experiences which would provide a deeper understanding of the factors contributing to delayed presentation and help inform context-specific interventions to improve timely care.

**********

what does this mean?). If published, this will include your full peer review and any attached files.

If you choose “no”, your identity will remain anonymous but your review may still be made public.

**Do you want your identity to be public for this peer review?**  For information about this choice, including consent withdrawal, please see our Privacy Policy

Reviewer #1: No

Reviewer #2: No

**********

[NOTE: If reviewer comments were submitted as an attachment file, they will be attached to this email and accessible via the submission site. Please log into your account, locate the manuscript record, and check for the action link "View Attachments". If this link does not appear, there are no attachment files.]

To ensure your figures meet our technical requirements, please review our figure guidelines: https://journals.plos.org/plosone/s/figures

You may also use PLOS’s free figure tool, NAAS, to help you prepare publication quality figures: https://journals.plos.org/plosone/s/figures#loc-tools-for-figure-preparation.

NAAS will assess whether your figures meet our technical requirements by comparing each figure against our figure specifications.

# Author comment: Author response to Decision Letter 1

**DOI:** 10.1371/journal.pone.0357668.r002

14 Aug 2026

A point-by-point response addressing all comments and suggestions from the Academic Editor and the two reviewers has been uploaded as an attachment ('Response to Reviewers').

Thank you

Attachment Submitted filename: Response to reviewers and academic editor.docx

# Editor report: Decision Letter 1

**DOI:** 10.1371/journal.pone.0357668.r003

**Authors:** Ozlem Boybeyi

20 Aug 2026

Management Outcomes and Factors Related to Hospital Stay for Pediatric Intussusception in a Tertiary Care Hospital, Northwestern Ethiopia

PONE-D-26-16101R1

Dear Dr. Temesgen,

We’re pleased to inform you that your manuscript has been judged scientifically suitable for publication and will be formally accepted for publication once it meets all outstanding technical requirements.

Within one week, you’ll receive an e-mail detailing the required amendments. When these have been addressed, you’ll receive a formal acceptance letter and your manuscript will be scheduled for publication.

An invoice will be generated when your article is formally accepted. Please note, if your institution has a publishing partnership with PLOS and your article meets the relevant criteria, all or part of your publication costs will be covered. Please make sure your user information is up-to-date by logging into Editorial Manager at Editorial Manager® and clicking the ‘Update My Information' link at the top of the page. For questions related to billing, please contact billing support.

If your institution or institutions have a press office, please notify them about your upcoming paper to help maximize its impact. If they’ll be preparing press materials, please inform our press team as soon as possible -- no later than 48 hours after receiving the formal acceptance. Your manuscript will remain under strict press embargo until 2 pm Eastern Time on the date of publication. For more information, please contact onepress@plos.org.

Kind regards,

Ozlem Boybeyi

Academic Editor

PLOS One

Additional Editor Comments (optional):

The authors have comprehensively revised the manuscript and have addressed both the knowledge gap and the public health relevance. The revisions to the main text and tables are also appropriate and sufficient, and they have improved the clarity and academic quality of the manuscript.

Given that the study addresses an important and clinically meaningful issue, particularly from a global public health perspective, I recommend the manuscript for publication in its current form.

However, in accordance with the journal’s authorship policy, it is recommended that the authors confirm that approval has been obtained from all authors regarding the change in authorship.

Reviewers' comments:

# Editor report: Acceptance letter

**DOI:** 10.1371/journal.pone.0357668.r004

**Authors:** Ozlem Boybeyi

PONE-D-26-16101R1

PLOS One

Dear Dr. Temesgen,

I'm pleased to inform you that your manuscript has been deemed suitable for publication in PLOS One. Congratulations! Your manuscript is now being handed over to our production team.

At this stage, our production department will prepare your paper for publication. This includes ensuring the following:

* All references, tables, and figures are properly cited

* All relevant supporting information is included in the manuscript submission,

* There are no issues that prevent the paper from being properly typeset

You will receive further instructions from the production team, including instructions on how to review your proof when it is ready. Please keep in mind that we are working through a large volume of accepted articles, so please give us a few days to review your paper and let you know the next and final steps.

Lastly, if your institution or institutions have a press office, please let them know about your upcoming paper now to help maximize its impact. If they'll be preparing press materials, please inform our press team within the next 48 hours. Your manuscript will remain under strict press embargo until 2 pm Eastern Time on the date of publication. For more information, please contact onepress@plos.org.

You will receive an invoice from PLOS for your publication fee after your manuscript has reached the completed accept phase. If you receive an email requesting payment before acceptance or for any other service, this may be a phishing scheme. Learn how to identify phishing emails and protect your accounts at https://explore.plos.org/phishing.

If we can help with anything else, please email us at customercare@plos.org.

Thank you for submitting your work to PLOS One and supporting open access.

Kind regards,

PLOS One Editorial Office Staff

on behalf of

Professor Ozlem Boybeyi

Academic Editor

PLOS One
